# Synthesis of Fe-Modified g-C_3_N_4_ Nanorod Bunches for the Efficient Photocatalytic Degradation of Oxytetracycline

**DOI:** 10.3390/ma17112488

**Published:** 2024-05-22

**Authors:** Dongmei Zhao, Xinyao Wang, Libin Wang, Jingzhen Wang, Xu Wang, Weipeng Cheng

**Affiliations:** 1 Department of Food, Heilongjiang East University, Harbin 150066, China; wl721328@sina.com; 2 School of Chemical Engineering and Materials, Heilongjiang University, Harbin 150080, China; wanglibin0927@163.com (L.W.); wjz13263546703wjz@163.com (J.W.); wx199900932@163.com (X.W.); chengweipeng12@163.com (W.C.)

**Keywords:** g-C_3_N_4_, Fe, chemical vapor co-deposition, photocatalytic degradation, oxytetracycline

## Abstract

Antibiotic residues have been found to have potentially harmful effects on ecological and human health. Carbon nitride-based photocatalysts have widely focused on antibiotic photocatalytic degradation. Herein, we prepared Fe-modified g-C_3_N_4_ nanorod bunches (FCNBs) using chemical vapor co-deposition. Specifically, through the process of calcination, a blend of urea and chlorophyllin sodium iron salt underwent an intriguing transformation, resulting in the integration of Fe into the framework of the g-C_3_N_4_ nanorod cluster. The resulting photocatalyst exhibited remarkable stability and superior dispersibility. The prepared FCNBs had a unique structure, which was beneficial for increasing light absorption. Furthermore, the Fe species formed a chemical coordination with the g-C_3_N_4_ matrix, thereby altering the electronic structure of the matrix. This modification facilitated charge transfer, prolonged the carrier lifetime, and enhanced light absorption, all of which significantly increased the photocatalytic activity. The oxytetracycline degradation efficiency of FCNBs was 82.5%, and they demonstrated outstanding stability in cycle trials. This work introduces a promising photocatalyst for the degradation of antibiotics.

## 1. Introduction

Oxytetracycline (OTC), an antibiotic with strong antibacterial action, has been extensively utilized in aquaculture, animal husbandry, and human and animal medicine in recent years [1,2,3,4]. Excessive OTC is discharged into wastewater or soil, causing harm to ecosystems and human health [5]. The ubiquity of OTC in water severely pollutes the water supply and induces microbial resistance. More and more attention has been paid to adopting effective methods to eliminate OTC in water. Currently, a multitude of methods, including physical adsorption, chemical oxidation, biodegradation, and photocatalytic degradation, have been employed for the elimination of OTC [6,7,8]. Among them, the photocatalytic degradation of OTC has attracted wide attention because of its green and sustainable characteristics. In principle, the photocatalytic degradation of oxytetracycline involves the selection of appropriate semiconductors and solar energy as the input to promote a chemical reaction through four main steps, as follows: 1. The catalyst needs to absorb photons whose energy is greater than or equal to the semiconductor band gap to achieve the necessary conditions for photogenerated electron and hole separation. 2. Electrons are excited from the valence band (VB) of the semiconductor to the conduction band (CB). 3. Optically excited charge carriers migrate to the surface without futile recombination. 4. The accumulated photoexcited electrons and holes participate in the redox reaction on the surface to realize the degradation of oxytetracycline [2]. The key to photocatalytic degradation technology is the rational design of a photocatalyst to improve the photocatalytic degradation ability.

As an N-type semiconductor, graphitic carbon nitride (g-C_3_N_4_) has led to great progress in boosting the photocatalytic degradation of oxytetracycline, estrone hormone, and dye and water splitting due to its visible-light response and excellent physicochemical properties [2,9,10,11,12]. However, there is still a need for improvement regarding its flaws, including the short visible-light absorption range, quick photogenerated carrier recombination, and low conductivity [10]. Doping, heterostructure construction, dye sensitization, and morphology control have been used to optimize g-C_3_N_4_ to enhance its photocatalytic performance [13,14,15,16]. Significantly, morphology regulation has been regarded as a simple and effective modification strategy. Jun et al. developed mesoporous g-C_3_N_4_, which enhances its ability to absorb light across a wider spectrum and extends the duration of the photoexcited charge carriers [17]. Similarly, Dou et al. prepared g-C_3_N_4_ nanosheets, significantly enhancing its photocatalytic degradation ability [18]. However, further alterations to its electronic structure are necessary in order to augment the optical, conductive, and other physical characteristics of g-C_3_N_4_.

The electronic structures of g-C_3_N_4_ can be successfully improved, and its photocatalytic degradation performance can be enhanced, by adding the highly conductive metal Fe [19]. Furthermore, lone pairs of electron, which can combine with Fe to generate Fe-N, are abundant in g-C_3_N_4_ [20]. Xu et al. utilized a one-step calcination technique for the production of Fe-doped g-C_3_N_4_ and this synthesis exhibited notable efficacy in the degradation of TC under visible-light-driven photodegradation reactions [21]. To enhance photocatalytic activity by encouraging the separation of electron–hole pairs, Sudrajat et al. incorporated Fe into g-C_3_N_4_ [22]. Van et al. used a simple and economical heat-stirring technique to create Fe-doped g-C_3_N_4_ high-performance photocatalysts [23]. However, these methods lead to the formation of Fe-particle-modified g-C_3_N_4_, which reduces the dispersion of Fe.

Herein, urea and a combination of chlorophyll sodium iron salt were utilized as precursors to create the uniformly distributed, Fe-modified, g-C_3_N_4_ nanorod bunches (FCNBs), which were then used to break down OTC under irradiation with visible light. The outcomes demonstrated the great stability and superior photocatalytic degradation performance of FCNBs, indicating their potential utility in the removal of organic pollutants such as OTC.

## 2. Experimental Section

### 2.1. Materials

Sinopharm Chemical Reagent Co., Ltd. (Shanghai, China) provided urea, ethanol, isopropanol (IPA), and ethylenediaminetetraacetic acid disodium (EDTA-2Na). Macklin Reagent Co., Ltd. (Shanghai, China) supplied chlorophyllin sodium iron salt, while benzoquinone (BQ) and OTC were procured from Shanghai Aladdin Biochemical Technology Co., Ltd. (Shanghai, China). We used all analytical-grade chemical reagents directly, without any purification.

### 2.2. Preparation of FCNBs and g-C_3_N_4_

Firstly, the filter paper made from cellulose was cut to a specific size and placed inside a nitrogen muffle furnace. Cellulose-based carbon paper was produced by gradually heating it at a rate of 3 °C/min in a nitrogen environment until it reached 700 °C, where it was held for 1 h. Afterwards, a mixture of 50 g urea and 0.5 g chlorophyll sodium iron salt was dissolved in a combination of 100 mL ethanol and deionized water at a temperature of 95 °C, resulting in the formation of a dark green solution. This solution was then heated under agitation for 2 h to obtain an evenly distributed solid mixture. The cellulose-based carbon paper was positioned on top of an alumina crucible, with its bottom covered by 10 g of the prepared mixture. Subsequently, the crucible underwent heating in a nitrogen atmosphere at a rate of 3 °C/min until reaching up to 550 °C for 2 h. As a result, the iron-modified g-C_3_N_4_ material adhered to the surface of the carbon paper, which is referred to as FCNBs. The FCNB on the carbon paper surface was collected into the sample tube by brushing. g-C_3_N_4_ was obtained by heating 20 g urea up to 550 °C at an identical heating rate under nitrogen atmosphere for 2 h.

### 2.3. Characterizations

The samples underwent analyses via the utilization of a Hitachi S-4800 scanning electron microscope (SEM) and a Japan Electronics Co., Ltd. (Amagasaki, Japan) JEM-2100 transmission electron microscope (TEM) to characterize their surface morphology and internal structure. Furthermore, functional groups of the composites were assessed through testing, analyzed using a Perkin-Elmer Spectrum One Fourier transform infrared spectrometer (FT-IR) that was equipped to operate within a range of 500–4000 cm^−1^. Measurements for X-ray diffraction (XRD) patterns were taken using a Bruker D8 Advance X-ray diffractometer, scanning within a 2θ range of 5–80° at a rate of 5°/min. The chemical state of Fe in the FCNBs, as well as the surface element composition and corresponding content distribution, were determined by VG ESCALAB 250Xi (Thermo Fisher Scientific, Waltham, MA, USA) X-ray photoelectron spectroscopy (XPS). To measure the composite material’s light absorption range, a Perkin-Elmer Lambda 950 UV-Vis spectrophotometer was utilized for analyses within a measuring range of 200–800 nm. Fluorescence intensity was measured using an FLS 920 fluorescence spectrophotometer (PL) to assess changes in the recombination efficiency of electron–hole pairs before and after modification, with an excitation wavelength set at 360 nm and measurement range from 300 to 700 nm. The Fe content in the samples was quantified using an Agilent 720 inductively coupled plasma optical emission spectrometer (ICP-OES).

### 2.4. Photoelectrochemical Measurements

Utilizing the CHI 760E electrochemical workstation, photoelectrochemical measurements were conducted. The working electrode was fabricated through the attachment of a sample to indium tin oxide glass. A platinum electrode served as the opposing pole, while the reference electrode, Ag/AgCl, was utilized. The utilized electrolyte was a 0.5 M Na_2_SO_4_ solution, with a 300 W xenon lamp serving as the illumination source. Analyses of the transient photocurrent response and electrochemical impedance spectroscopy were subsequently performed.

### 2.5. The Performance Measure of Catalysts

To assess how well the prepared samples degrade OTC under visible light, we dispersed 30 mg of the catalyst into 100 mL of a 10 mg/L OTC aqueous solution. To simulate visible light (λ > 420 nm), we used a 300 W xenon lamp (PLS-SXE300UV, Beijing Perfectlight Technology Co., Ltd., Beijing, China). Before irradiation, we stirred the mixture in darkness for 30 min to achieve an adsorption–desorption equilibrium. Throughout the irradiation process, we collected samples from the reaction solution at specific time intervals and then filtered them to remove the catalyst. We determined the OTC concentration by measuring its absorbance at 354 nm using a TU-1901 spectrophotometer. Additionally, we collected, washed, and dried samples for cycling experiments to evaluate the catalyst’s stability.

## 3. Results and Discussion

### 3.1. Characterization of Catalysts

**Figure 1:**
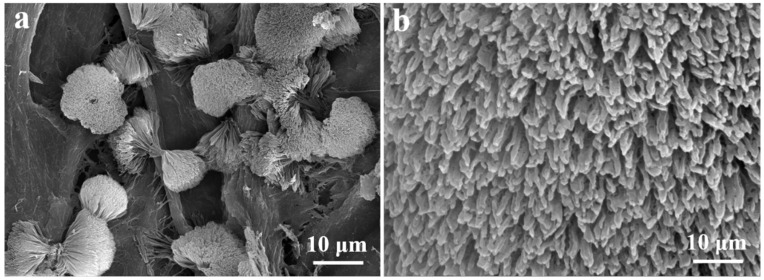
SEM image (**a**) and the magnified SEM image (**b**) of FCNBs.

**Figure 2:**
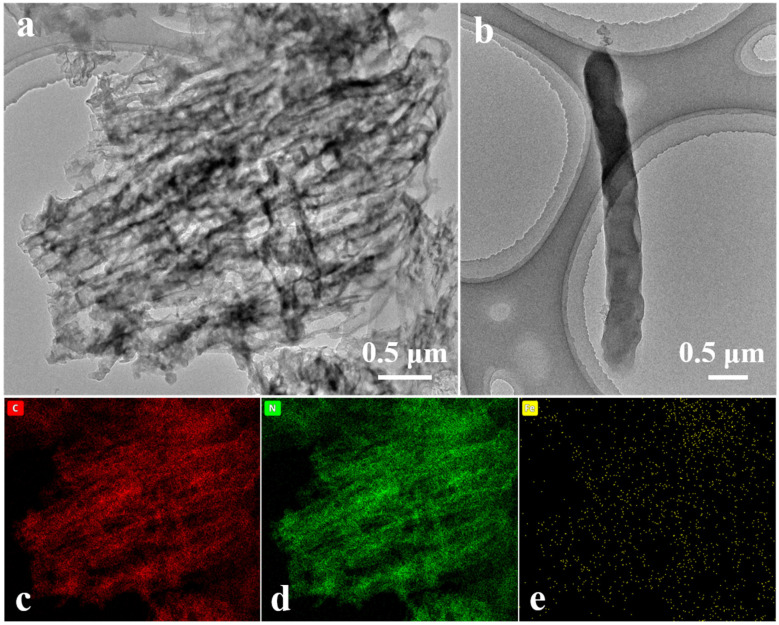
TEM image (**a**), the magnified TEM image of a single structural unit (**b**), and elemental mapping (**c**–**e**) of FCNBs.

**Scheme 1:**
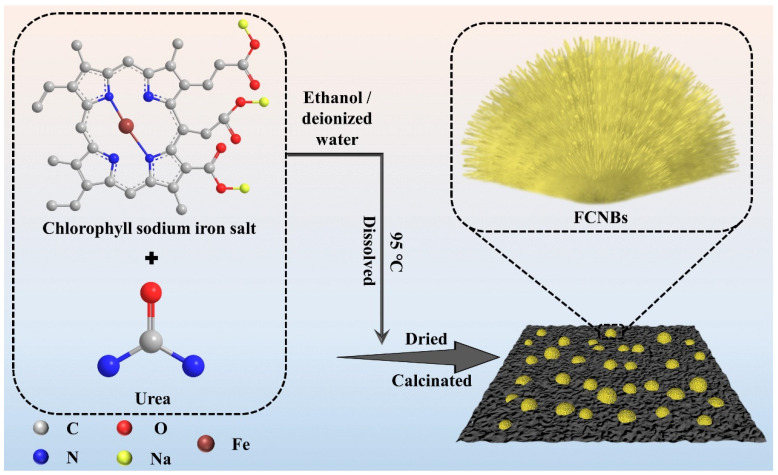
Flow chart for the synthesis of FCNBs.

FCNBs were prepared through chemical vapor co-deposition (Scheme 1). SEM and TEM were used to examine the shape and structure of FCNBs. As depicted in Figure 1 and Figure S1, FCNBs exhibit a radial bundle structure, which can be further confirmed by TEM. Figure 2 illustrates the rod-like structural unit of FCNBs, which has a diameter of around 350 nm. Through the corresponding EDS (Figure 2c–e), it can be judged that its constituent elements contain C, N, and Fe. The g-C_3_N_4_ boasts a high concentration of N atoms, encompassing six lone pairs of electrons. This unique molecular structure proves to be highly conducive to the bonding of iron, thereby introducing Fe into g-C_3_N_4_. The iron content of FCNBs was measured using ICP-OES and found to be 0.6 wt.% (Table S1), and it presented a uniform distribution across the entire surface of the g-C_3_N_4_.

**Figure 3:**
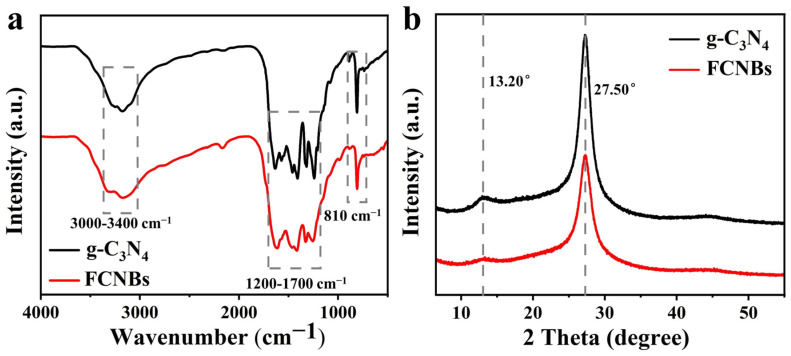
FT-IR spectra (**a**) and XRD spectra (**b**) of g-C_3_N_4_ and FCNBs.

Using XRD and FTIR spectroscopy, the materials’ structural and chemical characteristics were examined. Figure 3a displays the FTIR spectra of FCNBs and g-C_3_N_4_. The vibration peaks corresponding to the triazine unit vibration, stretching vibration of CN, and stretching vibration of N-H in free amino groups were located at 810 cm^−1^, 1200–1700 cm^−1^, and 3000–3400 cm^−1^, respectively [24]. g-C_3_N_4_ and FCNBs showed similar characteristic peaks, indicating that trace amounts of Fe did not significantly alter the basic structure of g-C_3_N_4_. The XRD patterns depicted in Figure 3b reveal the spectra of g-C_3_N_4_ and FCNB. Both catalysts show two distinct peaks at around 13.20° and 27.50°, associated with the stacking structure of the conjugated aromatic system and the in-plane structure of the tris-s-triazine unit, respectively [25]. Compared with g-C_3_N_4_, the diffraction peaks of FCNBs are generally weaker, which can be attributed to the interaction between the Fe and CN layers. In addition, no distinct diffraction peaks corresponding to elemental iron or iron oxide were observed, suggesting that the iron content may be very low or present in a coordinated form.

**Figure 4:**
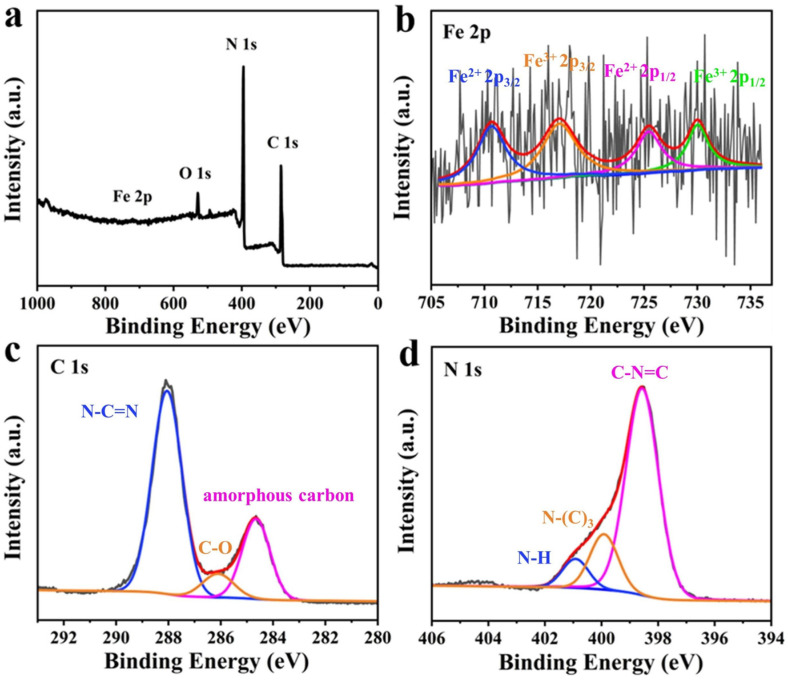
XPS survey spectra of FCNBs (**a**) and high-resolution XPS spectra of Fe 2p (**b**), C 1s (**c**), and N 1s (**d**).

The XPS analysis of FCNBs revealed a diverse surface elemental composition, as depicted in Figure 4a. Elements including C, N, O, and Fe were identified. The presence of O may be attributed to the adsorption of O_2_ or H_2_O on the composite surface [26]. Despite the low doping content of iron, distinct peaks corresponding to the iron species were not evident. The Fe 2p high-resolution spectrum (Figure 4b) exhibited peaks at 725.44 and 710.63 eV, indicative of the 2p_1/2_ and 2p_3/2_ states of Fe^2+^, respectively. Similarly, peaks at 730.01 and 717.05 eV corresponded to the 2p_1/2_ and 2p_3/2_ states of Fe^3+^ [23]. This observation confirms the coexistence of Fe^2+^ and Fe^3+^ in FCNBs. Figure 4c shows the high-resolution C 1s spectrum, which showed three peaks at 284.67, 286.11, and 288.05 eV. The peak at 284.67 eV represents amorphous carbon, while the peak at 286.11 eV indicates the presence of C-O bonds. The peak at 288.05 eV was associated with sp^2^-hybridised carbon in the N-C=N structure of the triazine ring [27]. In addition, Figure 4d shows the high-resolution N 1s spectrum with three distinct peaks. The peaks at 398.5, 399.9, and 400.92 eV correspond to sp^2^-bonded N atoms (C-N=C), tertiary nitrogen atoms in N-(C)_3_ groups, and N-H groups, respectively [28]. The tabulated form of elemental analysis from XPS is shown in Table S2 to confirm the element ratio of FCNB. We also conducted nitrogen adsorption and desorption tests on FCNB and g-C_3_N_4_. As shown in Table S3, the specific surface area of FCNB is slightly smaller than that of g-C_3_N_4_, which can be attributed to the introduction of Fe nanoparticles, leading to the destruction of the layered structure of g-C_3_N_4_ [26].

**Figure 5:**
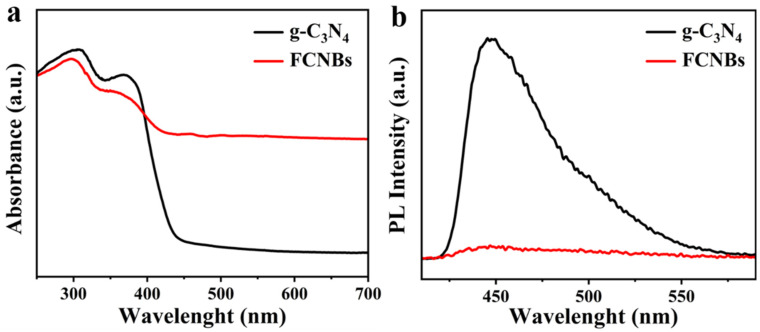
UV-vis diffuse reflectance spectra (**a**) and steady-state photoluminescence spectra (**b**) of g-C_3_N_4_ and FCNBs.

Figure 5a depicts the UV-Vis absorption spectra of g-C_3_N_4_ and FCNBs. Although g-C_3_N_4_ absorbs light across the ultraviolet and visible-light ranges, its absorption in the visible region is relatively weak. In contrast, FCNBs exhibited significantly improved absorption properties in the visible-light range due to the incorporation of Fe. Furthermore, there is a noticeable shift towards longer wavelengths in the absorption edge of FCNBs compared to that of g-C_3_N_4_, indicating that the addition of Fe can greatly enhance the capacity to absorb visible light. The improvement in the efficiency of solar energy utilization and boosting of the visible-light catalytic activity of the catalyst are the benefits of this enhancement. The evaluation of the photocatalytic performance of the photocatalysts was primarily determined by their efficiency in separating photogenerated electron–hole pairs. Therefore, we carried out photoluminescence (PL) testing. Figure 5b shows that g-C_3_N_4_ has a significant fluorescence peak at 450 nm, indicating a high rate of electron–hole pair recombinations after irradiation. In contrast, the fluorescence intensity of FCNBs notably decreased, suggesting that the incorporation of Fe can effectively suppress carrier recombination and enhance photocatalytic activity.

**Figure 6:**
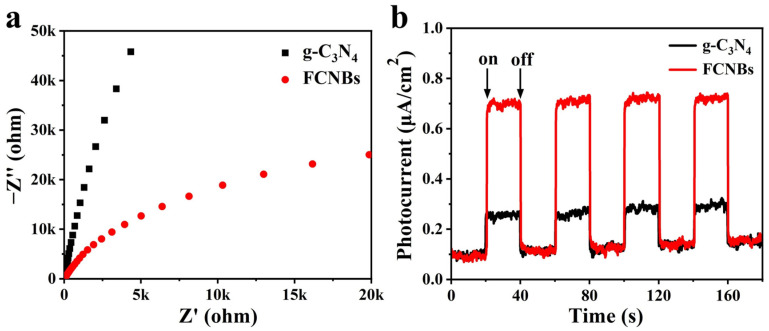
EIS Nyquist plots (**a**) and transient photocurrent density response (**b**) of nanosized and g-C_3_N_4_ and FCNBs.

We carried out an in-depth study of the light-induced electron transfer process and enhanced photocatalytic activity using electrochemical impedance spectroscopy (EIS) and photocurrent testing. The objective of this study is to explore the charge transfer resistance and efficiency in photogenerated electron–hole pairs’ separation. A comparison of the Nyquist plots of g-C_3_N_4_ and FCNBs in Figure 6a reveals that the arc radius of FCNBs is significantly smaller, the charge transfer resistance is reduced, and the electron transfer rate is accelerated. In addition, Figure 6b illustrates that the photocurrent intensity of FCNBs surpasses that of g-C_3_N_4_, indicating a substantial enhancement in the separation of the photogenerated electron–hole pairs.

### 3.2. Photocatalytic Activity Measurement

**Figure 7:**
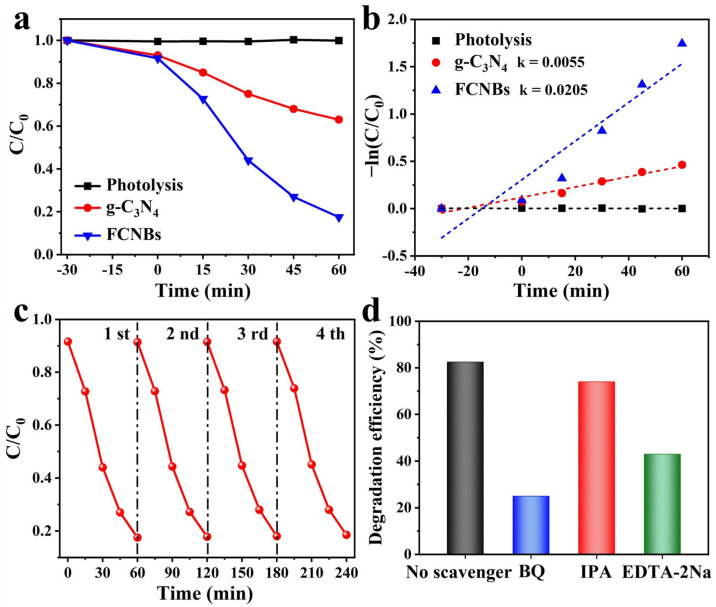
Photocatalytic activities (**a**) and kinetic fit (**b**) of the g-C_3_N_4_ and FCNBs for OTC photodegradation; (**c**) cycle runs for OTC photodegradation on FCNBs; (**d**) effect of different scavengers on the photodegradation of OTC compared to FCNBs.

The study investigated the degradation of OTC in the presence of visible light using the prepared materials. Figure 7a illustrates the OTC photodegradation process for different photocatalyst systems. Prior to visible-light irradiation (300 W xenon lamp), a dark reaction was conducted for 30 min to ensure catalyst adsorption saturation. The figure clearly demonstrates that, in the absence of any photocatalyst, OTC photodegradation is minimal, indicating the high stability of OTC under visible light. After 60 min of visible-light irradiation, g-C_3_N_4_ only degraded 37% of the OTC. In contrast, FCNBs achieved a photocatalytic efficiency of 82.5% within the same timeframe, which is superior to the similarly reported g-C_3_N_4_ catalyst at present (Table S4). This indicates that the introduction of iron notably augmented the photocatalytic properties of the composites. We increased the amount of FCNB from 30 to 50 mg to conduct a photodegradation experiment, and compared the effects of different amounts of the catalyst on the photodegradation performance. As shown in Figure S2a, the OTC degradation efficiency of 50 mg FCNB within 60 min is 84.9%, indicating that the degradation efficiency increases with an increase in the amount of catalyst. In addition, we carried out an in-depth study of the prepared samples to analyze the reaction kinetic properties of the photocatalytic degradation of OTC and applied Equation (1) to calculate the apparent rate constant (*k*) [29]:

*ln* (*C*_0_/*C*) = *kt* (1)

where *k* is the apparent rate constant, *t* is the light irradiation time, *C* is the concentration at irradiation time *t*, and *C*_0_ is the concentration at adsorption equilibrium.

The plot of *ln*(*C*_0_/*C*) versus time (*t*) demonstrates linearity in the kinetic analysis (Figure 7b), affirming that its degradation process adheres to first-order kinetics. The *k* for g-C_3_N_4_ is calculated as 0.0055 min^−1^. In contrast, the *k* value for FCNBs was determined to be 0.0205 min^−1^, which is 3.7 times greater than that of g-C_3_N_4_, underscoring the significantly enhanced degradation rate of the composite photocatalyst. In practical applications, the reusability and stability of composite materials are paramount. Consequently, cycling tests for OTC photocatalytic degradation were conducted on FCNBs to assess their reusability. In addition, we tested the photocatalytic degradation of OTC under the full solar spectrum with other conditions remaining unchanged. The results show that the photodegradation efficiency of the sample is 85.0%, as shown in Figure S2b. As depicted in Figure 7c, after four cycles, the degradation efficiency of the catalyst exhibited no notable decline, highlighting the excellent stability of FCNBs during photocatalytic degradation. We carried out SEM and XRD tests on the materials after the degradation test, as shown in Figure S3a,b. The considerable photodegradation efficiency of OTC makes FCNB an excellent material for the photocatalytic degradation of OTC.

### 3.3. Photocatalytic Mechanism

To delve deeper into the photocatalytic degradation mechanism, active radical trapping experiments were performed by using hydroquinone (BQ) and isopropanol (IPA) as a trap for the superoxide anion radical (•O^2−^) and hydroxyl radical (•OH), and disodium ethylenediamine tetraacetate (EDTA-2Na) as a hole-trapping agent [30]. We conducted an in-depth study on the role of these actives in the photocatalytic degradation process. As seen in Figure 7d, the introduction of IPA had no discernible impact on the degradation rate of OTC, suggesting that •OH had an insignificant effect on the degradation of OTC. However, the addition of BQ (10 mM) and EDTA-2Na (10 mM) led to a significant decrease in the degradation rate of OTC, highlighting the key role of •O^2−^ and h^+^ in the degradation process.

Furthermore, the Tauc diagram (Figure S4a) of g-C_3_N_4_ and FCNBs was obtained through UV-vis spectrum calculations, revealing band gaps of 2.92 eV and 2.48 eV for g-C_3_N_4_ and FCNBs, respectively. By combining this with the Mott–Schottky curves (Figure S4b,c), the band structure of g-C_3_N_4_ and FCNBs can be deduced (Figure S4d). The conduction band potential (E_CB_) of FCNBs is −0.55 V vs. NHE, which is lower than the O_2_/•O^2−^ potential (−0.33 V vs. NHE). This indicates that the photoelectrons on the CB of FCNBs are conducive to the reduction in •O^2−^. The valence band potential (E_VB_) of FCNBs is 1.93 V vs. NHE, which is lower than that of H_2_O/•OH (2.69 V vs. NHE). It can be inferred that holes (h^+^) cannot oxidize OH^−^ to •OH but directly oxidize OTC instead, based on these findings. This suggests that the influence of •OH on photocatalytic performance is negligible, consistent with the results of the capture experiment [31].

**Figure 8:**
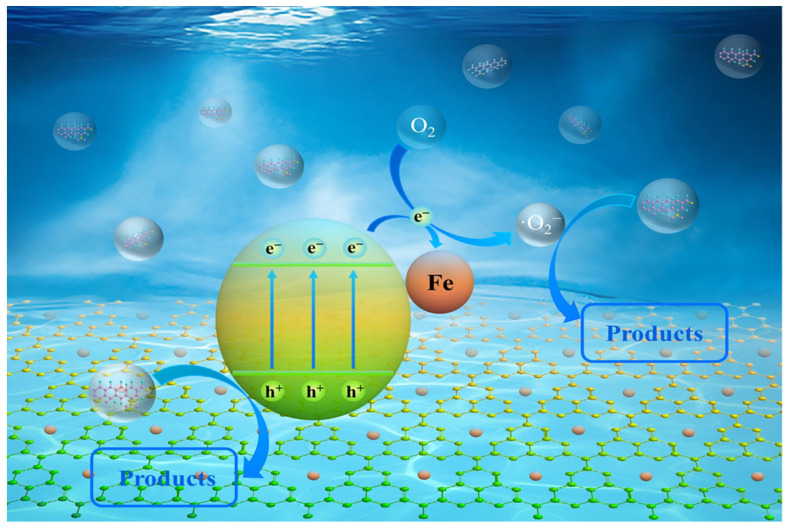
The possible photocatalytic mechanism of FCNBs for oxytetracycline degradation.

To explain how FCNBs degrade OTC, a tenable photocatalytic process is put forth (Figure 8). Under the given light conditions, electrons in the g-C_3_N_4_ material move to the conduction band through the valence band and then migrate to the iron state, improving the separation efficiency of electron–hole pairs. The h^+^ remaining in the valence band can oxidize contaminants. Subsequently, the photogenerated electrons may be captured by oxygen molecules to generate a potent oxidizing species •O_2_^−^, which decomposes antibiotics into smaller molecular species. Additionally, the incorporation of iron considerably boosts the absorption of visible light and enhances solar utilization. In conclusion, the improved photocatalytic degradation performance of FCNBs can be attributed to the increased light harvesting and higher electron–hole pair separation efficiency.

### 3.4. Possible Degradation Pathway of OTC by FCNBs

**Figure 9:**
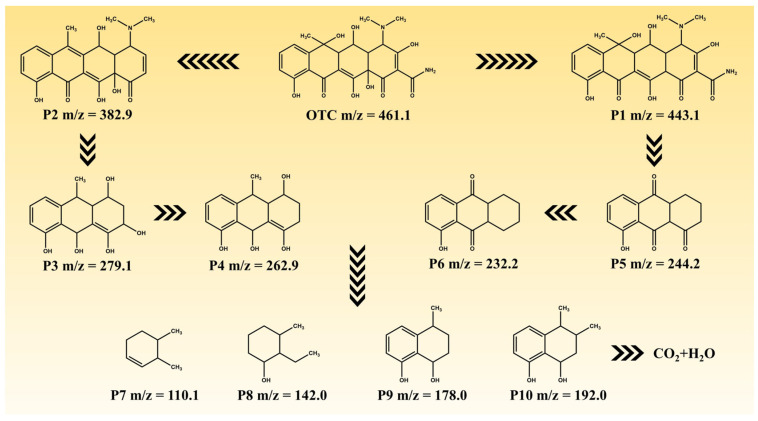
The possible degradation pathways of OTC.

The degradation intermediates of OTC were detected using LC-MS in order to elucidate its degradation pathway. Figure S5 and Table S5 present the potential intermediates, while Figure 9 illustrates two plausible main degradation pathways. In the first pathway, OTC undergoes dehydroxylation and deamidation, resulting in the formation of P2 (*m*/*z* = 382.9), which is subsequently further degraded to P3 (*m*/*z* = 279.1) through fragmentation. Further dehydroxylation leads to the generation of P4 (*m*/*z* = 262.9). In the second pathway, dehydration converts OTC into P1 (*m*/*z* = 443.1), which then decomposes and fragments into P5 (*m*/*z* = 244.2) via deamidation, demethylation, and dehydration processes. Subsequently, P5 is converted to P6 (*m*/*z* = 232.2) through deoxidation reactions. Finally, both P4 and P6 undergo additional decomposition steps, yielding lower-molecular-weight substances such as P7 (*m*/*z* = 110.1), P8 (*m*/*z* = 142.0), P9 (*m*/*z* = 178.0), and P10 (*m*/*z* = 192.0). These small molecules eventually degrade into CO_2_ and H_2_O after undergoing further degradation steps.

## 4. Conclusions

Graphene carbon nitride materials modified with iron were successfully synthesized using a homogeneous mixture of urea and chlorophyll iron sodium salt via chemical vapor-phase co-precipitation. Fe is uniformly dispersed within the internal structure of g-C_3_N_4_ through chemical coordination with the g-C_3_N_4_ matrix. Furthermore, Fe functions as an electron trap, thereby improving the separation efficiency of the generation of electron–hole pairs through light. The composite demonstrates efficient and stable photocatalytic degradation activity during the decomposition of OTC, making it a promising material for mitigating antibiotic contamination in the environment.

## Data Availability

Data are contained within the article and supplementary materials.

## Supplementary Materials

The following supporting information can be downloaded at: https://www.mdpi.com/article/10.3390/ma17112488/s1, Figure S1: SEM image of FCNBs; Figure S2: Photocatalytic activities of (a) 50 mg FCNBs, (b) 30 mg FCNBs in full solar spectrum of light; Figure S3: (a) XRD image and (b) SEM image of FCNBs after photocatalytic activity measurement; Figure S4: (a) Tauc curves of FCNBs and g-C_3_N_4_, Mott-Schottky curves of (b) g-C_3_N_4_ and (c) FCNBs, (d) the band structure of g-C_3_N_4_ and FCNBs; Figure S5: MS spectra of OTC solution after 60 min degradation; Table S1: Iron content of FCNBs; Table S2: Element content (wt.%) from XPS; Table S3: Specific surface area and pore parameter information of g-C_3_N_4_ and FCNBs; Table S4: Comparison of photocatalytic performance of photocatalysts based on g-C_3_N_4_ for degradation of OTC; Table S5: Structural information of the possible intermediates. Refs. [32,33,34,35] can be found in Supplementary Materials
