# Supplementary material for: Synthesis of Fe-Modified g-C3N4 Nanorod Bunches for the Efficient Photocatalytic Degradation of Oxytetracycline
Source: Materials (Basel). 2024 May 22;17(11):2488. doi: 10.3390/ma17112488 (PMC11172613; doi:10.3390/ma17112488)
Supplement: Supplementary file 1 [file materials-17-02488-s001.zip › materials-2969799-supplementary.pdf]

Supplementary Materials

Synthesis of Fe-Modified g-C_3N_4 Nanorod Bunches for the Efficient Photocatalytic Degradation of Oxytetracycline

Dongmei Zhao [1,*], Xinyao Wang [1], Libin Wang [2], Jingzhen Wang [2], Xu Wang [2] and Weipeng Cheng [2]

1 Department of Food and Environment Engineering, Heilongjiang East University, Harbin 150066, China; wl721328@sina.com
2 School of Chemical Engineering and Materials, Heilongjiang University, Harbin 150080, China; wanglibin0927@163.com (L.W.); wjz13263546703wjz@163.com (J.W.); wx199900932@163.com (X.W.); chengweipeng12@163.com (W.C.)
* Correspondence: zhaodongmei202205@163.com; Tel.: +86-158-4658-9441

Table S1. Iron content of FCNBs.

Sample	Fe (mas.%) from ICP-OES
FCNBs	0.6

Table S2. Element content (wt.%) from XPS.

Photocatalyst	C	N	O	Fe
g-C_3N_4	40.76	55.82	3.42	-
FCNBs	40.10	52.57	6.47	0.66

Table S3. Specific surface area and pore parameter information of g-C_3N_4 and FCNBs.

Photocatalyst	BET surface area ($m^2 \cdot g^{-1}$)	Pore volume ($cm^3 \cdot g^{-1}$)	Pore diameter (nm)
g-C_3N_4	73.731	0.718	3.934
FCNBs	56.860	0.514	3.714

Table S4. Comparison of photocatalytic performance of photocatalysts based on g-C_3N_4 for degradation of OTC.

Photocatalyst	Catalyst (mg)	Degradant ($mg \cdot L^{-1}$)	Efficiency (%)	K value (min^{-1})	Light source	Ref.
MIL-53Fe@g-C_3N_4	50	40 (100 mL) OTC	67 (60 min)	0.0178	300 W (halogen lamp)	[35]
CVC-2	100	20 (100 mL) OTC	80.5 (80 min)	0.014	40 W white LED	[36]
BPC(5%)/g-C_3N_4	50	10 (50 mL) OTC	84 (150 min)	0.00884	300 W (Xe lamp)	[37]
ZnO/g-C_3N_4	20	10 (100 mL) OTC	63.5 (50 min)	-	300 W (Xe lamp)	[38]
FCNBs	30	10 (100 mL) OTC	82.5 (60 min)	0.0205	300 W (Xe lamp)	This work

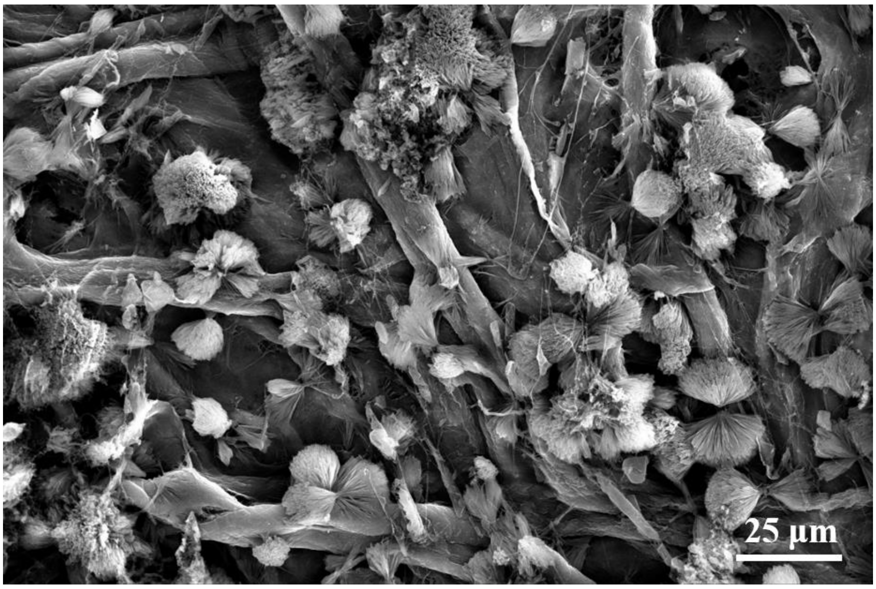

Figure S1. SEM image of FCNBs.

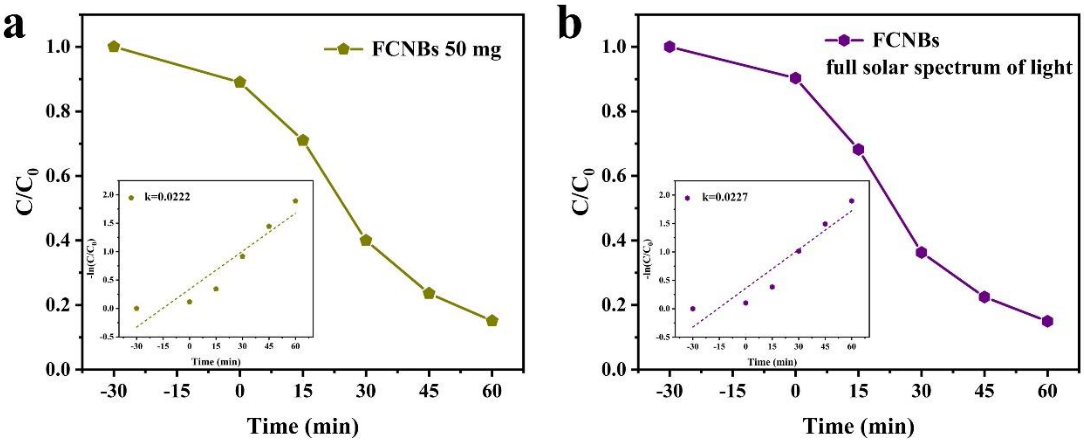

Figure S2. Photocatalytic activities of (a) 50 mg FCNBs, (b) 30 mg FCNBs in full solar spectrum of light.

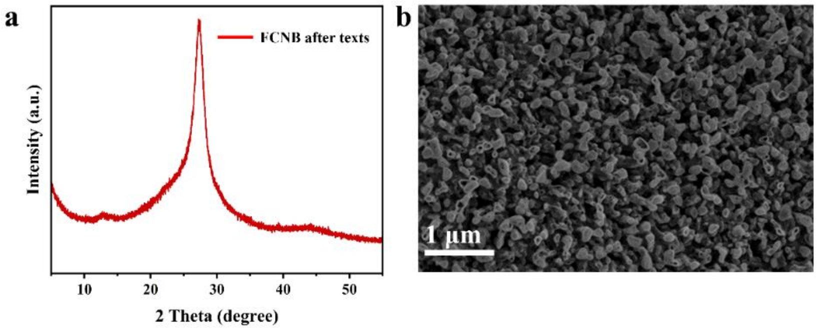

Figure S3. (a) XRD image and (b) SEM image of FCNBs after photocatalytic activity measurement.

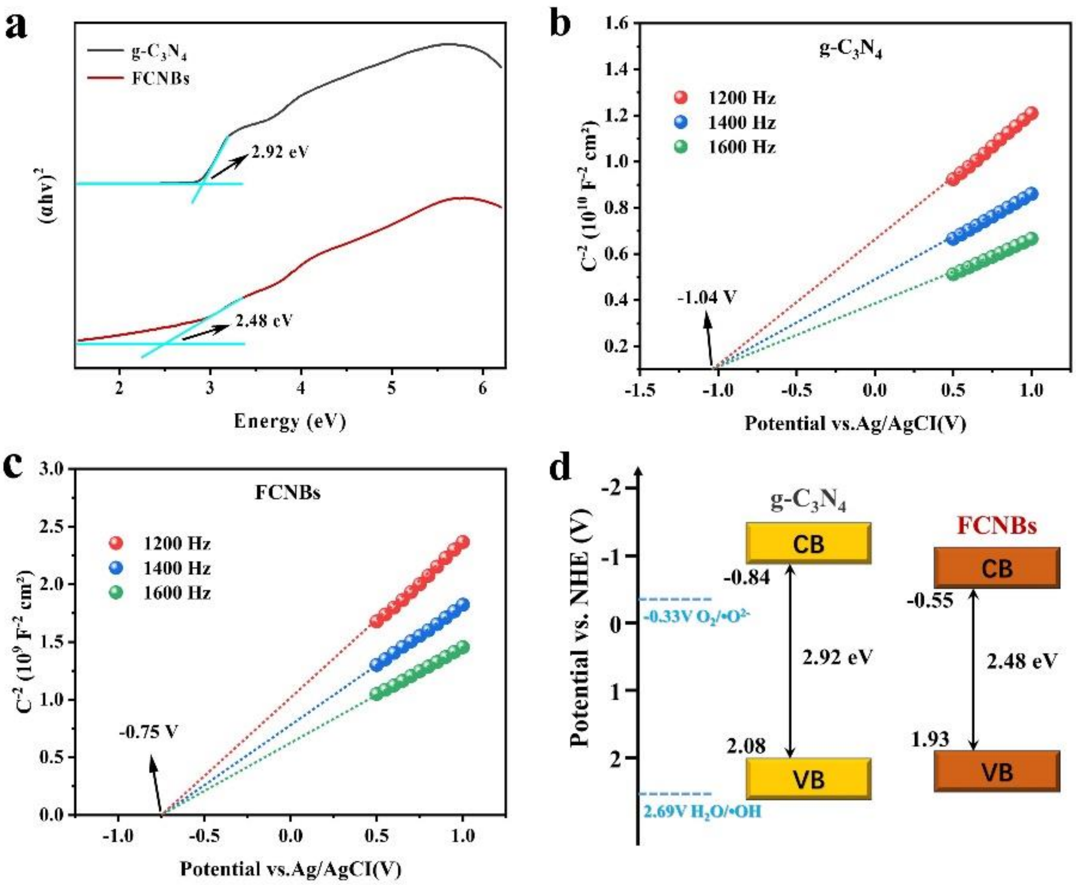

Figure S4. (a) Tauc curves of FCNBs and g-C_3N_4, Mott-Schottky curves of (b) g-C_3N_4 and (c) FCNBs, (d) the band structure of g-C_3N_4 and FCNBs.

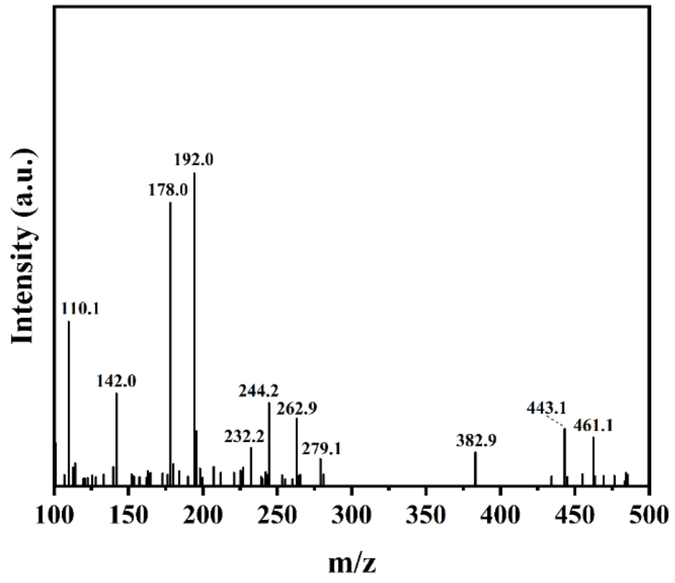

Figure S5. MS spectra of OTC solution after 60 min degradation.

Table S5. Structural information of the possible intermediates.

Compounds	Formula	m/z	Proposed structure
**OTC**	$C_{22}H_{24}N_2O_9$	461.1	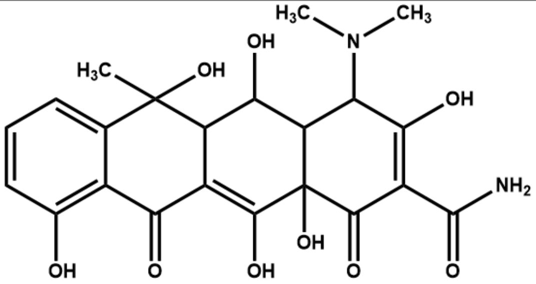
**P1**	$C_{22}H_{22}N_2O_8$	443.1	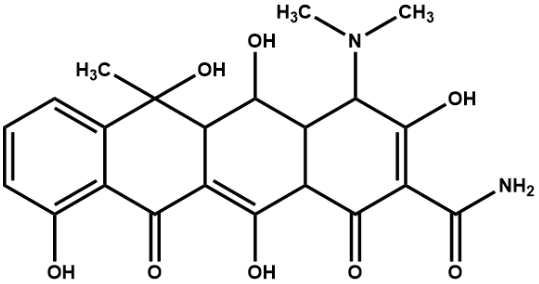
**P2**	$C_{21}H_{21}NO_6$	382.9	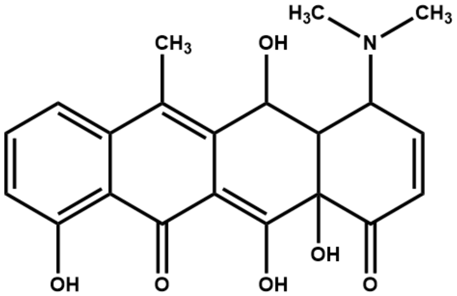
**P3**	$C_{15}H_{18}O_5$	279.1	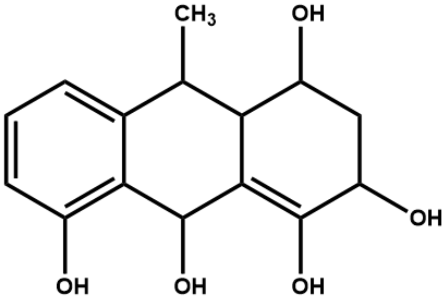
**P4**	$C_{15}H_{18}O_4$	262.9	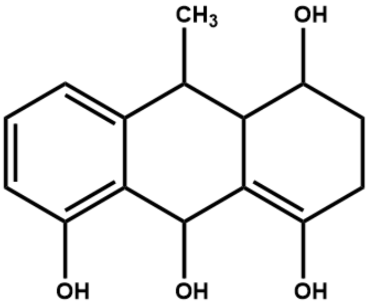
**P5**	$C_{14}H_{12}O_4$	244.2	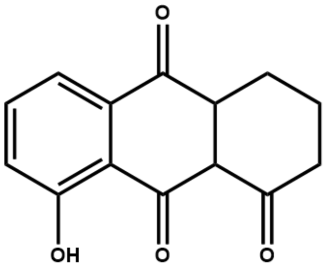

**P6**	$C_{14}H_{16}O_3$	232.2	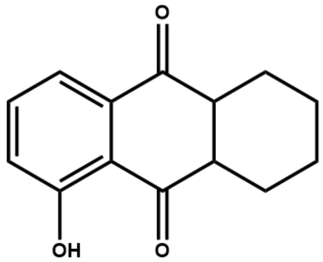
**P7**	C_8H_{14}	110.1	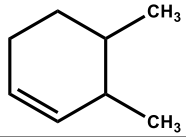
**P8**	$C_9H_{18}O$	142.0	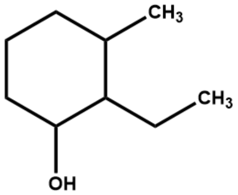
**P9**	$C_{11}H_{14}O_2$	178.0	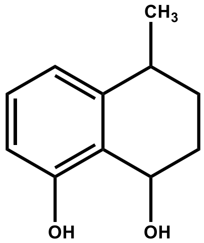
**P10**	$C_{12}H_{16}O_2$	192.0	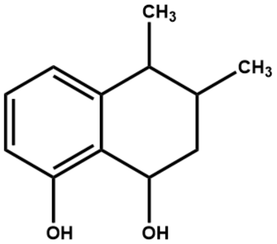
